# Repetitive nociceptive stimulation elicits complex behavioral changes in *Hirudo*: evidence of arousal and motivational adaptations

**DOI:** 10.1242/jeb.245895

**Published:** 2023-08-15

**Authors:** Jessica Hoynoski, John Dohn, Avery D. Franzen, Brian D. Burrell

**Affiliations:** Division of Basic Biomedical Sciences, Center for Brain and Behavioral Research (CBBRe), Sanford School of Medicine, University of South Dakota, Vermillion, SD 57069, USA

**Keywords:** Nociception, Pain, Arousal, Sensitization, Invertebrate, Leech

## Abstract

Appropriate responses to real or potential damaging stimuli to the body (nociception) are critical to an animal's short- and long-term survival. The initial goal of this study was to examine habituation of withdrawal reflexes (whole-body and local shortening) to repeated mechanical nociceptive stimuli (needle pokes) in the medicinal leech, *Hirudo verbana*, and assess whether injury altered habituation to these nociceptive stimuli. While repeated needle pokes did reduce shortening in *H. verbana*, a second set of behavior changes was observed. Specifically, animals began to evade subsequent stimuli by either hiding their posterior sucker underneath adjacent body segments or engaging in locomotion (crawling). Animals differed in terms of how quickly they adopted evasion behaviors during repeated stimulation, exhibiting a multi-modal distribution for early, intermediate and late evaders. Prior injury had a profound effect on this transition, decreasing the time frame in which animals began to carry out evasion and increasing the magnitude of these evasion behaviors (more locomotory evasion). The data indicate the presence in *Hirudo* of a complex and adaptive defensive arousal process to avoid noxious stimuli that is influenced by differences in internal states, prior experience with injury of the stimulated areas, and possibly learning-based processes.

## Teaser

**Highlighted Article:** Repeated noxious stimulation of the medicinal leech elicits a transition from reflexive withdrawal to pre-emptive evasion behaviors that is modifiable as a result of differences in internal state or prior injury.

## INTRODUCTION

Pain is a critical sensory process that helps to protect an animal from and/or minimize its exposure to damage or potentially damaging stimuli. As defined by the International Association for the Study of Pain (IASP; https://www.iasp-pain.org/publications/iasp-news/iasp-announces-revised-definition-of-pain/), pain has both a sensory (nociceptive) component and an emotional/affective component. In addition to acute responses, prior nociceptive events can produce changes in behavioral responses to subsequent stimuli. Classic examples include sensitization to nociceptive/painful (hyperalgesia) or non-nociceptive/non-painful (allodynia) stimuli (Lewin and Moshourab, 2004), but there are also changes that involve a decrease in responses to pain, e.g. stress-induced analgesia or habituation to pain (Bingel et al., 2007; Butler and Finn, 2009; De Paepe et al., 2019). These relatively simple stimulus–response level changes likely contribute to more complicated changes in behavior following noxious stimuli, such as changes in prey hunting, predator avoidance, sheltering decisions, conditioned place avoidance (to avoid conditions associated with previous injury) and conditioned place preference (preference for conditions associated with pain relief) (Alupay et al., 2014; Appel and Elwood, 2009; Crook, 2021; Crook et al., 2014, 2011). All of these pain-based behavioral changes are thought to be adaptive and promote the animal's continued health and survival (Walters and Williams, 2019; Williams, 2016).

Our group has used the medicinal leech, *Hirudo verbana*, to study neuromodulation in nociceptive and non-nociceptive synapses and the impact of such modulation on behavior (Hanson and Burrell, 2018; Jorgensen and Burrell, 2022; Paulsen and Burrell, 2019; Wang and Burrell, 2018; Yuan and Burrell, 2013). *Hirudo* possess a complement of somatic sensory neurons similar to those found in vertebrates (Smith and Lewin, 2009). These include rapidly adapting touch (T) cells, slow-adapting pressure (P) cells, and both mechanical and polymodal nociceptor (N) cells (Blackshaw et al., 1982; Burrell, 2017; Nicholls and Baylor, 1968; Pastor et al., 1996). In addition, the central nervous system (CNS) of *Hirudo* is well characterized and highly accessible to electrophysiological recording, lending itself to a wide variety of neurophysiological and neuroethological studies (Kristan et al., 2005; Wagenaar, 2015).

The initial intent of this study was to examine habituation of withdrawal reflexes to nociceptive stimuli and the effects of prior injury on nociceptive habituation. Habituation is a non-associative form of learning in which responses to repeated stimuli decrease in a manner that is not the result of fatigue or sensory adaptation (Groves and Thompson, 1970; Rankin et al., 2009). Studies of habituation to painful stimuli in humans report increases in the threshold to painful stimuli and decreases in pain ratings, pain-evoked reflexes, and neurological signals associated with pain (Bingel et al., 2007; Breimhorst et al., 2012; Ernst et al., 1986; Milne et al., 1991). However, the capacity to habituate to pain may be easily disrupted (De Paepe et al., 2019) and there are reports of deficits in habituation in some chronic pain conditions, e.g. migraines and fibromyalgia (Coppola et al., 2013; Smith et al., 2008).

To our surprise, while repeated nociceptive stimulation did produce a reduction of evoked withdrawal reflexes, we also observed additional behavioral changes in these animals. Specifically, *Hirudo* exhibited increasing levels of evasion behaviors (sucker evasion or locomotory evasion) that appeared to be initiated to avoid subsequent nociceptive stimuli. Furthermore, the transition from reflexive withdrawal to evasive behaviors appeared to be subject to differences in the internal state of the animal, which we propose to reflect an arousal state, and by the experience of a prior injury.

## MATERIALS AND METHODS

Medicinal leeches (*Hirudo verbana* Carena 1820) that weighed 2–3 g were used for these experiments (purchased from Leeches.com). Within the laboratory, the animals were housed in an incubator at 15°C in artificial pond water (0.5 g of Instant Ocean l^−1^ distilled water) on a 12 h light/dark cycle.

**Fig. 1.**
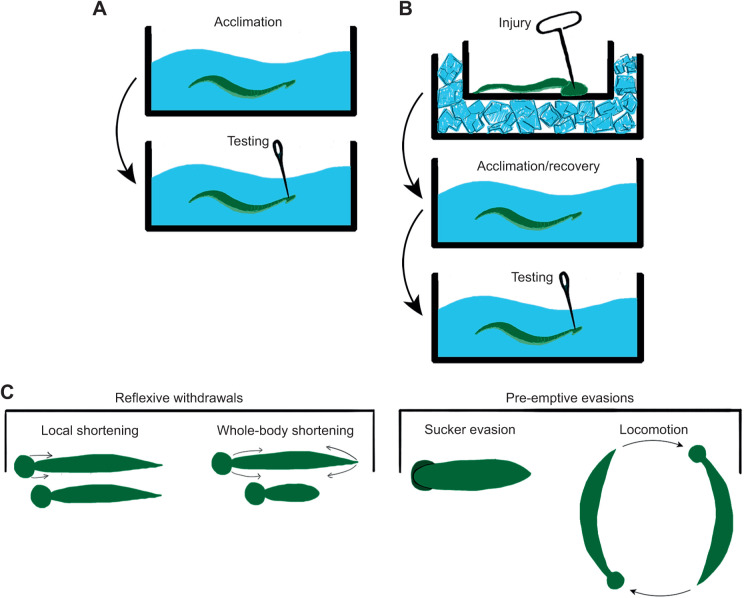
**Experimental protocols.** (A) *Hirudo verbana* were placed in the testing chamber (Petri dish) for 30 min prior to undergoing testing. During testing, 40 needle pokes at a 1 min intertrial interval were delivered to the posterior sucker. (B) In experiments that tested for the effects of injury, a T-pin was used to pierce the posterior sucker while the animals were lightly anesthetized in an ice-lined dish. The animals were then transferred to the testing chamber for 30 min followed by testing. (C) Four distinct behaviors were observed during testing. Local shortening or whole-body shortening in response to a needle poke, and then sucker evasion or locomotory evasion when the animal was due to be poked (digital art by J.D.).

*Hirudo verbana* were taken from the incubator and placed separately in a 145 mm diameter Petri dish filled with 35 ml of artificial pond water at room temperature (21–23°C). The dishes were covered with lids to prevent the animals from escaping. Each animal received a unique identifier consisting of the date and a number from 1 to 10 (e.g. 5.17 1) to keep track of those that were tested over multiple days. The animals were allowed to acclimate for 30 min before testing began (Fig. 1A). Animals were monitored during both the acclimation and testing periods with a video camera (Sony Handycam).

A 25-gauge needle was used to deliver repetitive mechanical nociceptive stimuli to the leeches as described in previous studies using rodents (Cowie et al., 2018; Hogan et al., 2004), which has been shown to be a noxious/sensitizing stimuli in *Hirudo* (Mack et al., 2022). Needle pokes were applied to the posterior sucker, which is easy to consistently target with repeated pokes in the same location (Fig. 1A). Stimulus duration was approximately 1 s and care was taken not to pierce the skin (no bleeding was observed). Forty stimuli were delivered at 1 min inter-stimulus intervals. In some experiments, animals were injured prior to the acclimation period (Fig. 1B). Animals were placed in an ice-lined dish for 3–5 min prior to injury, making them less mobile and easier to handle. The injury was induced with a T-pin (4.56 cm long, 1.0 mm diameter) to pierce the middle of the posterior sucker. In order to make the injuries relatively uniform between animals, the pin was inserted through the sucker and then quickly withdrawn while the leech was in ice-cold pond water. This minimized the opportunity for movement that might increase the size of the wound. Immediately after injury, *H. verbana* were transferred to the Petri dish with room temperature pond water. This approach has been shown to elicit a consistent level of sensitization to subsequent mechanical stimuli applied to the sucker lasting at least 7 days (Jorgensen and Burrell, 2022). Following the acclimation period in the Petri dish, the leeches received repetitive stimulation with the 25 gauge needle (Fig. 1B).

Behavioral responses to needle pokes could be readily categorized as either local shortening (scored as a 1) or whole-body shortening (scored as a 2; Fig. 1C). Local shortening involves the contraction of only a few body segments, usually the stimulated segment and adjacent segments (Kristan et al., 2005): in these experiments, withdrawal of just the posterior sucker and the adjacent 1–2 body segments. Whole-body shortening, in contrast, involves the near-simultaneous contraction of all the body segments. Local shortening and whole-body shortening are mediated by distinct neural circuits (Shaw and Kristan, 1995; Wittenberg and Kristan, 1992a,b). After only a few replicates of this experiment, it became clear that the repetitive mechanical stimulation was also producing a second category of behavioral responses that were mutually exclusive from the two types of shortening reflexes. These were referred to as evasion behaviors because they occurred prior to the delivery of the stimuli and allowed the animals to avoid a subsequent needle poke to the posterior sucker. The two evasion behaviors observed were either a persistent hiding of the posterior sucker under the body (sucker evasion=1) or locomotory behavior (locomotory evasion=2). In the context of these experiments, locomotion was usually crawling because the depth of the pond water in the Petri dish was too shallow to accommodate swimming. Evasion behaviors prevented the experimenter from applying a needle poke to the posterior sucker during that trial, but the experimenter still removed the lid of the Petri dish and placed the needle in the water in proximity to the animal during all 40 trials. In some experiments, animals were tested and then retested 7 days later, injured and then tested once 7 days later, or injured once and tested twice (on the day of injury and again 7 days post-injury). These animals were placed in individual jars with pond water and kept in the incubator at 15°C until the day of re-testing.

The responses for each of the 40 stimuli were assessed as either a reflexive withdrawal, with a score of 1 or 2 for local or whole-body shortening, respectively, or an evasion, with a score of 1 or 2 for sucker evasion or locomotory evasion, respectively. If there was neither an evasion nor a shortening response during a trial, then a score of 0 was recorded. Because the reflexive withdrawal and the evasion behaviors were mutually exclusive, it is possible to use the same scoring system for each behavior. The withdrawal or evasion scores across the 40 trials were binned into blocks of five trials each to facilitate analysis (that is, a score was averaged over five trials to produce a trial block score). All statistical and graphical analyses were carried out using GraphPad. Withdrawal or evasion score data are presented as means±s.e.m. These data were tested using one-way or two-way analysis of variance (ANOVA) statistics (the data passed GraphPad's within tests for normal distribution). We also counted the number of needle pokes an animal received to determine how many stimuli were required before evasion behaviors became the dominant response. Non-parametric statistical analyses were used on these data, either Mann–Whitney paired comparisons or Kolmogorov–Smirnov distribution statistics. The number of pokes and behavioral response categories were also analyzed using raster plot graphs.

## RESULTS

### Repetitive nociceptive stimulation elicits multiple behavioral adaptations

**Fig. 2.**
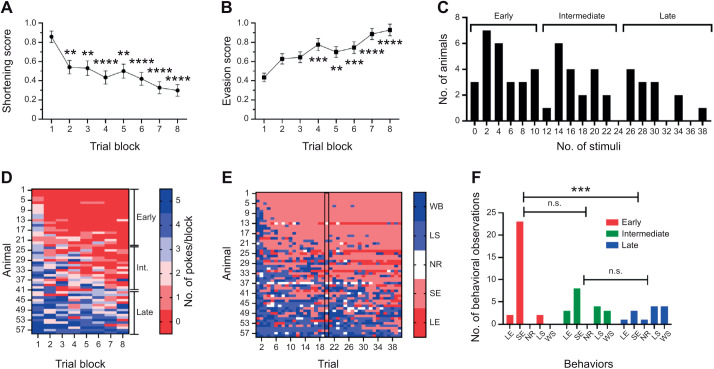
**Behavioral effects of repeated nociceptive stimulation.** (A) Shortening reflex scores decreased across trial blocks. (B) Evasion scores increased across trial blocks. Asterisks indicate a statistically significant difference relative to trial block 1. Asterisks indicate a significant difference based on one-way ANOVA (**P<0.005, ***P<0.001 and ****P<0.0001). (C) Frequency distribution of the number of pokes delivered during testing indicates how quickly evasion behaviors were initiated across the 58 animals tested. A multi-modal distribution was observed indicating the presence of three sub-populations that utilize a distinct behavioral strategy: early, intermediate and late evaders. (D) Raster plot showing the pattern of pokes per trial block for all 58 animals with the proposed early, intermediate and late evader designations indicated on the right. Red shades indicate fewer or no pokes per trial block and blue shades indicate more pokes per trial block. (E) Raster plot showing the distribution of shortening (blue shades) and evasion (red shades) responses over all 40 trials for each of the 58 animals tested. White indicates no response for that trial. The rectangle at trial 20 indicates the data used for the frequency distribution analysis in F. (F) Frequency distribution of behavioral responses for early, intermediate and late evaders during trial 20 (indicated by the rectangle in E). Asterisks indicate a significant difference in distribution based on Kolmogorov–Smirnov statistics (***P<0.001); n.s., not significant. LE, locomotory evasion; SE, sucker evasion; NR, no response; LS, local shortening; and WS, whole-body shortening.

Forty needle pokes at 1 min intervals were delivered to the posterior sucker (Fig. 1A) and the behavioral responses were categorized as local shortening, whole-body shortening or no response (Fig. 1C). The expectation was that the shortening reflex behavioral scores would decrease in the average responses for five trials binned into eight trial blocks (i.e. 8 trial blocks with 5 trials per block). Decreases in the shortening response score over the eight trial blocks were observed (Fig. 2A; one-way ANOVA, *F*=6.16, *P*<0.0001, *N*=58). However, this change was not due solely to a decrease in the magnitude of the shortening reflex, i.e. more local shortening responses or no-responses to needle pokes. Instead, an increased appearance of a new set of unstimulated behaviors that precluded poke-elicited shortening responses was observed. Specifically, animals began to persistently withdraw their posterior sucker so that it was hidden underneath the segments just anterior of the sucker or continuously locomote (crawl) within the Petri dish (Fig. 1C). These evasion behaviors were observed prior to the experimenter removing the Petri dish lid and placing the needle in the pond water and did not appear to be an enhanced response to visual or water motion stimuli associated with placing the needle in the water. This could mean that the behavior was initiated in the preceding trial and continued to the current trial or was initiated during the inter-trial period. These behaviors were referred to as sucker evasions and locomotory evasions, and the level of the evasion behavior score increased significantly across the trial blocks (Fig. 2B; *F*=7.45, *P*<0.0001, *N*=58).

We wanted to examine whether different animals had different criteria for whether to engage in reflexive shortening versus evasion. One way to assess this was to count the number of needle pokes that were delivered over the 40-trial testing period. Fewer pokes during the experiment indicated that the leeches quickly began to engage in evasion over shortening, while more pokes indicated that reflexive shortening persisted in these animals. A frequency distribution analysis of the number of needle pokes across 58 tested animals is shown in Fig. 2C. This revealed that the number of pokes was distributed into three distinct peaks or modes, indicating that the data had a multi-modal distribution. We confirmed that these data were not normally distributed using Shapiro–Wilks statistics (*W*=0.94, *P*<0.005). We propose that this multi-modal distribution is due to the presence of three populations of leeches with distinct behavioral strategies: early evaders that began evading following relatively few (<12) needle pokes, late evaders that persisted in reflexive shortening through the majority (25 or more) of the experimental trials, and intermediate evaders that received 12–23 needle pokes during testing. A comparison of the median and 95% confidence interval (CI) showed that there was no overlap in the CI between the three groups, supporting the conclusion that a multi-modal distribution of behavioral types was observed. The median (lower, upper 95% CI) was 4 pokes (2,7) for the early evaders, 16 (14,19) for the intermediates and 28 (25, 33) for the late evaders. To further test the idea that the categories described in Fig. 2C corresponded to temporally different behavioral strategies, we analyzed the distribution of pokes delivered over time (trial blocks) and the types of behavioral responses over time. First, a raster plot was generated in which the number of pokes per trial block (range 0–5) was plotted for each animal over the eight trial blocks (Fig. 2D). This analysis supported the idea that the number of pokes over testing corresponded to early, intermediate and late evaders. Those with the fewest pokes were placed at the top of the *y*-axis and those with the most were placed at the bottom. Animals poked relatively few times received those pokes almost entirely in trial block 1, while those poked the most had those stimuli distributed over all eight trial blocks.

To confirm that the categorizations based on the number of pokes corresponded to differences in the actual behavioral responses, a second raster plot plotted the behavioral scores for each animal across all 40 trials (Fig. 2E). Here, the scoring was changed so that reflexive shortening was scored with positive numbers (represented in blue), evasion behaviors were scored with negative numbers (represented in red), and no response to a poke was scored as 0 (represented in white). This raster plot shows that animals receiving relatively few pokes were engaged in evasion behaviors in earlier trials, while those receiving many pokes engaged in relatively few evasion behaviors even late in testing, supporting the categorization of animals as early, intermediate and late evaders. Next, data from trial 20 were extracted from animals in all three categories (indicated by the rectangle in Fig. 2E) for frequency distribution analysis (Fig. 2F). Data from trial 20 were shown because this represents the mid-point of the testing period. These data show that the animals previously categorized as early evaders exhibited far more evasion behaviors compared with the late evasion group and that the distributions between the two groups were statistically significant (Kolmogorov–Smirnov, *P*<0.005). No differences were observed in the behavioral distribution between the intermediate evasion group and the early or late evasion groups (*P*=0.23 and 0.49, respectively). Collectively, these data support the categorization of animals into early, intermediate and late evaders and indicate that there is considerable behavioral flexibility between individuals in their adaptation to repeated noxious stimuli.

Because the shortening and evasion behaviors were mutually exclusive (i.e. there can be no stimulus-evoked withdrawal when the animal is evading), this complicates assessing whether there were changes in the shortening reflexes with repeated stimulation. While a statistically significant decrease in the shortening scores was observed over the eight trial blocks (Fig. 2A), this response decrement is an artifact of the increase in evasion behaviors. A lack of response to the needle pokes is normally scored as a zero, but a poke cannot be applied to the posterior sucker when the animal is hiding the sucker or moving to avoid the poke; therefore, shortening cannot be elicited to test. To address this issue, we selectively examined shortening in leeches from the late evader category (>25 pokes; *N*=13) and only shortening behaviors during trial blocks 1–4. In this way, an assessment of changes in shortening behavior could be made that minimized the number of conflicting evasion behaviors (see Fig. 2E, animals 45–58). A decrease in the shortening response score across trials blocks 1–4 was observed in these animals, but it was not statistically significant (Fig. S1; one-way ANOVA, *F*=2.74, *P*=0.054).

**Fig. 3.**
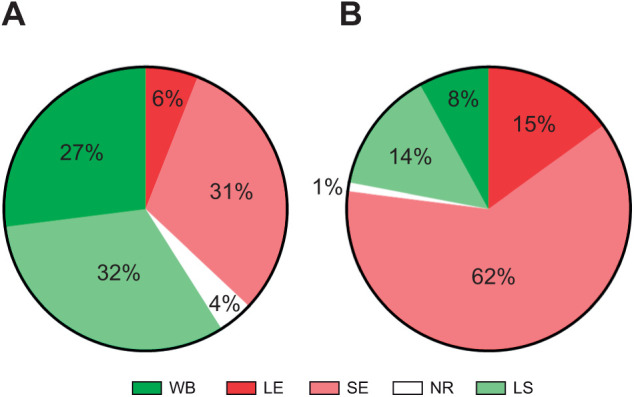
**Distribution of behaviors from all 58 animals tested.** (A) Non-injured trial block 1. (B) Non-injured trial block 8. LE, locomotory evasion; SE, sucker evasion; NR, no response; LS, local shortening; and WS, whole-body shortening.

To examine the changes in behavior during repeated nociceptive stimulation in more detail, the percentage of shortening and evasion behaviors employed by all animals during trial block 1 and trial block 8 was graphed (Fig. 3). During trial block 1, shortening reflexes represented the majority of the behaviors observed, although a substantial number of sucker evasion behaviors were observed as well (Fig. 3A). The appearance of evasion behaviors this early is consistent with the observations in Fig. 2C–F that a significant number of animals quickly adopted an evasion strategy, thus receiving fewer needle pokes. In trial block 8, evasion behaviors dominated, with sucker evasion being far more common than locomotory evasions (Fig. 3B). Relatively few local or whole-body shortening responses were observed in trial block 8 and these represent the contribution of the late evaders. In both blocks, there were very few cases when the animal failed to respond to stimulation (categorized as ‘no response’ in Fig. 3). Chi-square analysis did confirm a significant difference in the distribution of behaviors between block 1 and block 8 (χ=70.25, *P*<0.0001).

A portion of the 58 leeches from Fig. 2 underwent a second round of needle pokes 7 days later (*N*=10). The idea was to test whether the evasion scores as a group would be greater on day 7, potentially indicating a memory of the prior testing. No differences in the decrease in shortening behavioral scores (Fig. S2A) and increase in the evasion scores (Fig. S2B) between day 1 and day 7 were observed. For shortening, a two-way ANOVA detected a significant effect of trial block (*F*=4.32, *P*<0.005) but no significant effect of testing day (*F*=0.07, *P*=0.80) and no interaction effect (*F*=0.62, *P*=0.74). For evasion, the two-way ANOVA detected a significant effect of trial block (*F*=8.00, *P*<0.0001), but no effect of testing day (*F*=0.98, *P*=0.32) and no interaction effect (*F*=0.56, *P*=0.79). To summarize, one round of nociceptive stimulation did not alter an animal's response to a second round of stimuli, at least in the 7 day time frame that was tested.

### Effects of injury-adaptive behavioral changes

**Fig. 4.**
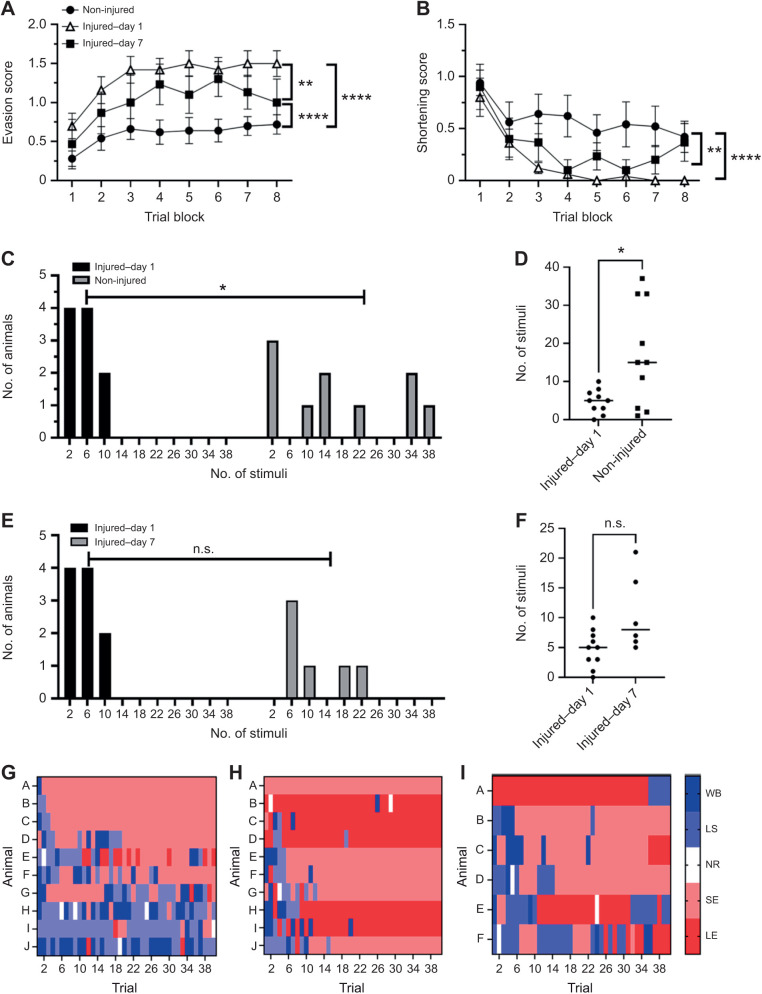
**Effect of injury on shortening and evasion behaviors.** (A) *H. verbana* tested on the same day as injury (injured–day 1) exhibited significantly greater evasion scores compared with non-injured animals. This effect persisted 7 days later (injured–day 7), although the increase in evasion scores was reduced compared with that for the injured–day 1 group. (B) Pattern of decreases in the shortening behavior scores between the three groups. (C) Injured–day 1 animals exhibited early-evader behavior based on the distribution of the number of needle pokes delivered in that group compared with the subset of non-injured animals analyzed. The distribution in the injured group was different from that of the non-injured group based on Kolmogorov–Smirnov analysis (see Results). (D) Injured–day 1 animals received fewer needle pokes (because they engaged in evasion earlier) compared with non-injured animals based on Mann–Whitney analysis. (E) The distribution of needle pokes in the injured–day 1 group was not different from that of the injured–day 7 group based on Kolmogorov–Smirnov analysis (see Results). (F) There was no difference in the number of needle pokes delivered between the injured–day 1 and injured–day 7 animals. (G–I) Raster plots comparing the distribution of shortening and evasion behaviors over time (trial) for the non-injured (G), injured–day 1 (H) and injured–day 7 (I) animals. (G) In non-injured animals, a range of early, intermediate and late evaders was observed. (H) In injured–day 1 animals, it appeared that only early evaders were observed. (I) In the injured–day 7 animals, most appeared to be early evaders with a small number of intermediate evaders. LE, locomotory evasion; SE, sucker evasion; NR, no response; LS, local shortening; and WS, whole-body shortening. Asterisks indicate a significant difference (*P<0.05, **P<0.005 and ****P<0.0001).

Next, we examined the effect of a prior injury on the pattern of putative evasion behaviors to repetitive nociceptive stimuli (Fig. 1B). In Fig. 4, three groups of animals were compared: a non-injured group consisting of 10 animals randomly selected from the 58 leeches tested in Fig. 2 (referred to as non-injured–day 1), an injured group of animals tested on the same day as injury (injured–day 1; *N*=10), and a group that was injured but not tested until 7 days later (injured–day 7; *N*=6). The injury consisted of a piercing of the posterior sucker using a T-pin, which has been validated in a previous *H. verbana* study as producing non-nociceptive sensitization that lasts 7 days as well as short-term (<1 day) nociceptive sensitization (Jorgensen and Burrell, 2022).

Injury significantly increased the evasive behavior scores on both day 1 and day 7 (Fig. 4A). Animals in all three groups exhibited an increase in their evasion scores with repetitive stimulation, which was confirmed by a two-way ANOVA detecting a significant effect of trial block (*F*=4.16, *P*<0.005). However, injury increased the magnitude of the evasion behaviors (indicated by the evasion score) during testing both on the same day as injury (injured–day 1) and 7 days later (injured–day 7). This was confirmed by a statistically significant effect of injury (*F*=37.85, *P*<0.0001). Furthermore, the injury effect on evasion behaviors was maximum on day 1 and decreased by day 7 but was still significantly different from the non-injured group. Tukey's *post hoc* analysis of the injury effect showed that the injured–day 1 group was significantly different from both the non-injured group (*P*<0.0001) and the injured–day 7 group (*P*<0.005). The injured–day 7 group was also different from the non-injured group (*P*<0.0001). The two-way ANOVA detected no significant interaction effect (*F*=0.32, *P*=0.99). The increase in the evasion scores suggests that the injured animals engaged in more locomotory evasion behaviors compared with the non-injured animals.

Decreases in the shortening behavior scores across trial blocks were observed in all three groups (Fig. 4B). Although there appeared to be a greater decrease in shortening in the injured groups compared with the non-injured group, again this is likely an artifact of the increased number of evasions in the injured–day 1 and injured–day 7 groups compared with the non-injured group.

Next, we wanted to examine the role that injury plays in the transition from reflexive shortening to evasion behaviors. As shown in Fig. 2C–E, *H. verbana* exhibited a multimodal distribution in terms of the number of needle pokes needed before switching to mostly evasion behaviors. Does injury affect that distribution? To answer this, we again plotted the frequency distribution of needle pokes for both the injured–day 1 and the non-injured group (Fig. 4C). Animals from the injured group received far fewer pokes across trial blocks compared with the non-injured group and this difference in distribution was confirmed by Kolmogorov–Smirnov analysis (*P*<0.05). The non-injured animals exhibited the same pattern of multimodal distribution observed in Fig. 2C (albeit with a sparser distribution because of the reduced sample size), which is not surprising given that this is a subset of 10 animals randomly selected from the 58 animals shown in Fig. 2C. As an additional test, analysis of the number of pokes delivered across all trial blocks showed that the injured–day 1 group received fewer needle pokes compared with the non-injured group (Fig. 4D; Mann–Whitney, *P*<0.05; non-parametric test used as there was a difference in the variance).

We also examined whether there were changes in the number of needle pokes received during testing between the two injured groups. In terms of the frequency distribution of the number of needle pokes, while the injured–day 7 group exhibited somewhat more spread compared with the injured–day 1 group (Fig. 4E), the difference in distribution between the two groups was not statistically significant (Kolmogorov–Smirnov, *P*=0.99). Non-parametric paired comparison of the number of pokes delivered also indicated no significant difference between the injured–day 1 and injured–day 7 groups (Fig. 4F; Mann–Whitney, *P*=0.051). Raster plot analysis of behavioral scores across trials supported the conclusion that injury shifted the distribution of behaviors in favor of the early evasion strategy, including considerably more locomotory evasion, compared with the non-injured group (Fig. 4G–I). This was also supported when these animals were analyzed in terms of the number of pokes per trial block (Fig. S3).

**Fig. 5.**
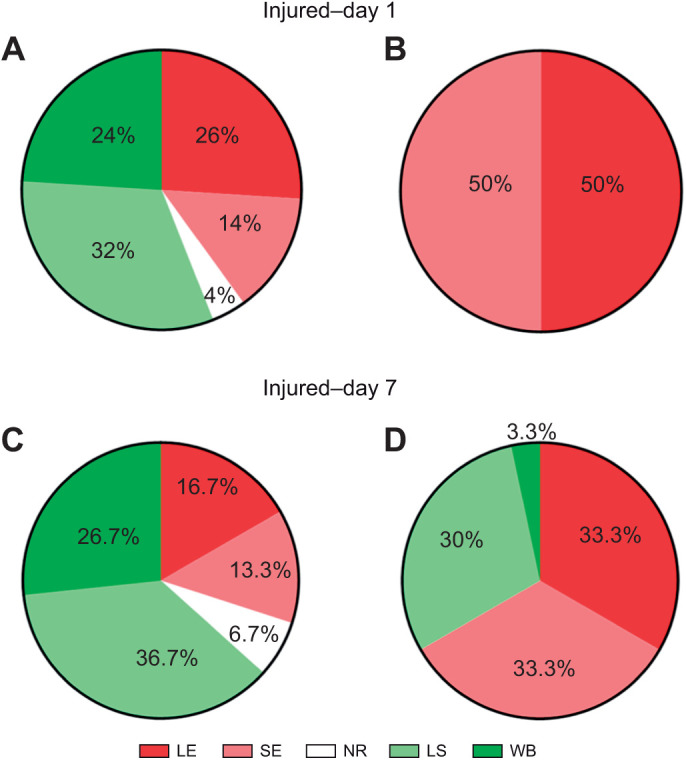
**Proportion of different reflexive shortening and persistent evasion behaviors observed in injured–day** **1 and injured–day** **7 groups.** (A) In injured–day 1 leeches during trial block 1, a mix of behaviors was observed, with the shortening reflexes being more predominant. (B) By trial block 8, only evasion behaviors were observed and there was an equal proportion of sucker evasion and locomotory evasion. (C) In injured–day 7 animals during trial block 1, a similar mixture of shortening and evasion behaviors was observed. (D) During trial block 8, these animals again exhibited more evasion behaviors, but shortening responses were also observed, unlike in the injured–day1 group. LE, locomotory evasion; SE, sucker evasion; NR, no response; LS, local shortening; and WS, whole-body shortening.

We again examined the changes in the percentage of shortening and evasion behaviors by all animals during trial block 1 and trial block 8, in injured–day 1 and injured–day 7 animals (Fig. 5). For the injured–day 1 animals, the distribution of shortening and evasion behaviors during trial block 1 was similar to that of the non-injured animals (compare Figs 5A and 3A). The local and whole-body shortening reflexes dominated during trial block 1, although a substantial number of evasion behaviors were observed as well. In trial block 8, however, only evasion behaviors were observed (Fig. 5B). Chi-square analysis did confirm a significant difference in the distribution of behaviors between block 1 and block 8 in this injured group (χ=174.7, *P*<0.0001). The complete lack of shortening response in block 8 is consistent with the observation that all injured–day 1 animals exhibited the early evader strategy (see Fig. 4C). In addition, the percentage of locomotory evasions during block 8 was substantially greater than what was observed in non-injured animals at this same time point. This is consistent with the observation that the evasion scores in the injured–day 1 animals were significantly greater over the eight trial blocks compared with the non-injured animals (Fig. 4A).

In the injured–day 7 animals, we again found that shortening behaviors dominated during trial block 1, comparable to the other injured and non-injured groups during this time period (Fig. 5C). In trial block 8, the injured–day 7 animals differ from the injured–day 1 animals (Fig. 5B) in that the day 7 animals exhibited a mixture of evasion and shortening behaviors (Fig. 5D). Nevertheless, chi-square analysis did confirm a significant different in the distribution of behaviors between block 1 and block 8 in this group injured and tested on day 7 (χ=39.39, *P*<0.0001). This is consistent with the observation that the evasion scores in the injured–day 7 group are significantly lower over the eight trial blocks compared with those of the injured–day 1 animals (Fig. 4A) and indicates that this decrease was due to fewer locomotory evasions and more shortening responses than observed in the day 1 group. The presence of more shortening responses in trial block 8 is also in agreement with the significant differences in shortening scores between the injured–day 1 and injured–day 7 groups (Fig. 4B). Together, these results suggest a partial recovery from injury over the 7 day period.

### Effect of injury plus retesting

**Fig. 6.**
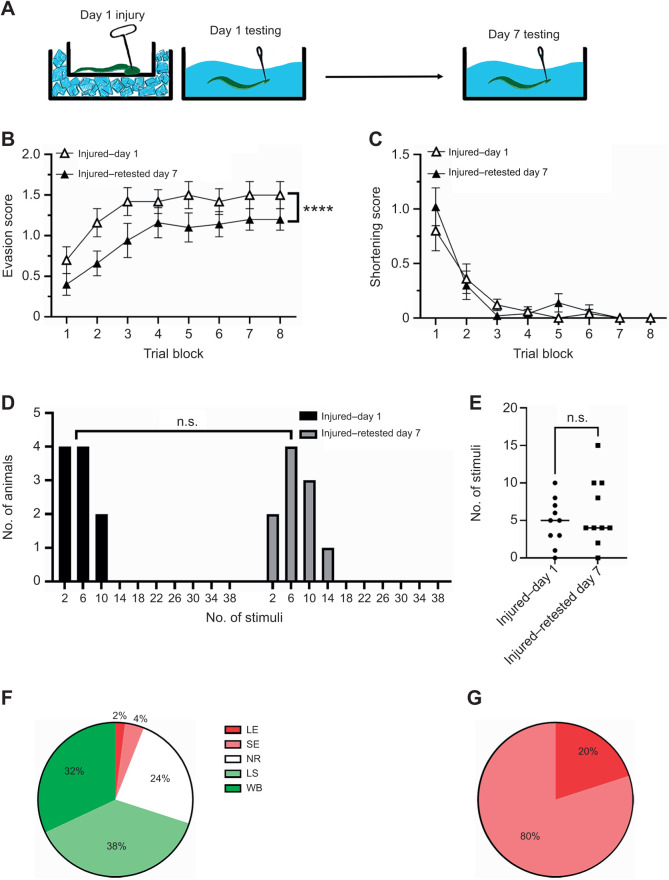
**Effects of injury plus retesting.** (A) *H. verbana* were injured and tested on day 1 and then retested on day 7. (B) The evasion scores were reduced in the injured–retested day 7 group compared with the injured–day 1 animals. (C) No differences were observed in the shortening reflex scores between the two groups. (D) No differences were observed in the frequency distribution of pokes delivered between the injured–day 1 and injured–retested day 7 groups. (E) There were no differences in the number of stimuli delivered on days 1 and 7. (F) In the injured–retested day 7 group during trial block 1, shortening behaviors dominated, but there were noticeably more ‘no responses’ and few evasion behaviors. (G) By trial block 8, only evasion behaviors were observed, with sucker evasion being the most prominent. LE, locomotory evasion; SE, sucker evasion; NR, no response; LS, local shortening; and WS, whole-body shortening.

Although retesting 7 days later had no effect on evasion scores or shortening responses (Fig. S2), we examined what would happen if *H. verbana* were retested in the context of injury (Fig. 6A). Here, animals from the injured–day 1 group were retested on day 7. As shown in Fig. 6B, the evasion responses from the injured–day 1animals were significantly greater compared with those of the same animals retested on day 7 (injured–retested day 7). Two-way ANOVA detected a significant effect of treatment group (tested day 1 versus retested day 7; *F*=18.55, *P*<0.0001), a significant effect of trial block (*F*=5.94, *P*<0.0001), but no interaction effect (*F*=0.17, *P*=0.99). The change in the evasion scores between day 1 and day 7 for these two groups appeared to be no different from what was observed in injured animals tested on day 7 for the first time (compare Fig. 4A with Fig. 6B). No differences due to testing day were observed in the shortening responses (Fig. 6C). Two-way ANOVA detected no significant effect of treatment group (day 1 versus day 7; *F*=0.34, *P*=0.56), a significant effect of trial block (*F*=26.04, *P*<0.0001), and no interaction effect (*F*=0.74, *P*=0.64). Frequency distribution analysis of the number of needle pokes on day 1 versus day 7 also revealed no statistically significant differences (Fig. 6D; Kolmogorov–Smirnov, *P*=0.99). There was also no difference in the number of pokes delivered on day 1 versus day 7 (Fig. 6E; Mann–Whitney *P*=0.67).

In terms of the distribution of evasion and shortening behaviors during trial blocks 1 and 8, there were some differences compared with the other experimental groups. During trial block 1, while the percentage of local and whole-body shortening responses was comparable to that of other groups, the level of evasion behaviors was substantially lower and the percentage of ‘no responses’ was substantially higher (Fig. 6F). During trial block 8, only evasion behaviors were observed, with sucker evasion being more prevalent than locomotory evasion (Fig. 6G). Chi-square analysis did confirm a significant difference in the distribution of behaviors between block 1 and block 8 in this injured and then re-tested on day 7 group (χ=177.5, *P*<0.0001). This is consistent with a decrease in the evasion scores between the injured–day 1 and injured–retested day 7 groups (Fig. 6B) and indicates that it was a reduction in the locomotory evasions that contributed to the lower scores. This is different from the injured–day 1 group, where trial block 8 was also dominated by evasion behaviors, but there was a much higher level of locomotory evasion behaviors (Fig. 6B). It is also different from the animals that were injured and tested for the first time 7 days after injury (injured–day 7 group; Fig. 6D). In that group, there were fewer evasion behaviors because of a higher level of shortening responses. The reason for these differences is unclear, but one possibility is that the retesting process promotes an increased number of evasions in the context of injury. Such a retesting effect may have counteracted the proposed recovery effect observed in the injured–day 7 animals. It is worth noting that the process of retesting non-injured animals had no effect on evasion or shortening behaviors (Fig. S2). This suggests that the capacity for retesting to promote evasion behaviors only occurs in the context of injury.

## DISCUSSION

Repeated delivery of needle pokes to the posterior sucker were found to elicit a surprisingly complex set of behavioral adaptations in *H. verbana*. Animals initially responded with reflexive shortening as expected, but these reflexive withdrawal responses were replaced by evasion behaviors (sucker evasion and locomotory evasion). We refer to these as evasion behaviors because they were initiated before the experimenter could deliver a needle poke on the next trial. *Hirudo* do have simple eyes and low-threshold mechanosensory cells for detecting water movement that could potentially be responding to mechanical stimuli associated with the needle entering the water or visual stimuli from the hand lifting the Petri dish lid or holding the needle (Friesen, 1984; Groves and Jellies, 2021; Stowasser et al., 2019). However, the animals were engaging in evasion behaviors throughout the inter-trial period or initiated during this period and not in response to the experimenter. The transition from transient reflexive responses (local and whole-body shortening) to more persistent avoidance behaviors (sucker and locomotory evasions; Figs 2D,E, and 3) may represent a behavioral assessment on the leech's part. That is, there is a benefit in avoiding further exposure to noxious stimuli that outweighs the costs of either keeping the posterior sucker hidden or engaging in constant locomotion. The increase in the time spent evading and the magnitude of the evasion behaviors (locomotory evasion>sucker evasion) may be comparable to escalations observed in other species in the context of predator avoidance, defensive behaviors or intraspecies aggression (Bubak et al., 2014; Crook et al., 2014, 2011; Gibson et al., 2015; Rillich and Stevenson, 2011). A caveat to this interpretation is that behavioral variability may have been due to other factors, e.g. experimenter variability in the application of the needle poke or differences between animals in the response properties of the periphery.

The combination of behavioral changes during repetitive needle pokes may represent a form of increased arousal in *H. verbana*. This is based on the observation that the evasion behaviors persisted beyond the period immediately after stimulus delivery (Anderson and Adolphs, 2014; Lebestky et al., 2009). Another element of arousal observed in these data is scalability, a continuum of increasing magnitude of behavioral responses. This was observed in the transition from local to whole-body shortening reflexes, which involves activation of motor neurons in an increasing number of body segments (Shaw and Kristan, 1995; Wittenberg and Kristan, 1992a). The sucker evasions and locomotory evasions may also represent an increasing scale of evasion responses. There was also evidence of variations in the arousal state, i.e. the distribution of the early, intermediate and late evaders in non-injured animals (Fig. 2C–F). It is not clear what mediates this variation in when to engage in evasion strategies, but *H. verbana* do possess a number of neurotransmitters associated with modulation of pain in mammals, and stress and arousal in diverse species including *Hirudo*, e.g. serotonin, dopamine and endocannabinoids (Gaudry and Kristan, 2009; Mesce et al., 2001; Paulsen and Burrell, 2019; Puhl and Mesce, 2008). Another form of modulation of arousal is the effect of prior injury, which changed the proportion of leeches that could be categorized as early evaders. Injured animals began evasion earlier and engaged in much more locomotory-based evasions compared with non-injured animals (Figs 4C,H and 5A). The injury effect persisted for at least 7 days, although there was evidence of partial recovery at this point (Figs 4I and 5D). One interpretation of these behavioral results is that prior injury changes the assessment of how quickly and to what extent the animal should engage in evasion behaviors to prevent additional damage to an already-injured part of the body. These results suggest that *H. verbana* has the capacity to recognize and assess its injured condition and adjust its aversive responses to noxious stimuli as observed in other vertebrates and invertebrates (Khuong et al., 2019; Zhang et al., 2017). Other changes in internal or external conditions might affect the transition to evasion behaviors, e.g. feeding state, the presence of potential prey or predators, or the presence of other leeches (Bisson et al., 2012; Gaudry and Kristan, 2009; Harley et al., 2011). These findings are significant because variability in responses to noxious stimuli, even when the stimuli intensity is constant, is thought to be due in part to differences in the motivational element of pain in humans (Auvray et al., 2010; De Paepe et al., 2019).

Initiating or continuing evasion behaviors without an explicit stimulus may also reflect a form of self-protective behavior analogous to wound tending, guarding, grooming and rubbing, further evidence of *H. verbana* being in an internally driven aversive state (Williams, 2016). The proposed presence of anticipatory behaviors to noxious stimuli in *H. verbana* is interesting because it is potentially similar to how motivation influences the perception of pain in humans (Auvray et al., 2010). The ability to selectively protect a specific body region has also been observed in the octopus (Crook, 2021) and such observations may indicate evidence of sensory-discriminative capabilities for noxious stimuli in invertebrates comparable to what is observed in human responses to painful stimuli (Auvray et al., 2010; Crump et al., 2022). *Hirudo* sensory neurons, including the nociceptors, have well-characterized receptive fields (Blackshaw et al., 1982; Johansen et al., 1984; Nicholls and Baylor, 1968) and these afferents have an impressive capacity to encode stimulus location (Baca et al., 2005; Lewis and Kristan, 1998; Pirschel and Kretzberg, 2016).

Alternatively, sucker evasions may represent sensitization of the local shortening reflex, expressed as a prolongation of the shortening duration. The increased persistence in locomotory evasion without the need for additional needle pokes may also involve sensitization. Examples of sensitization to a specific site on the body or a specific type of stimulus are widely observed in invertebrates and vertebrates, e.g. site-specific sensitization, intrinsic sensitization or pseudoconditioning (Burrell and Sahley, 1998; Davis and File, 1984; Erickson and Walters, 1988; Katz, 1998; Walters, 1987a,b, 1994). This does not contradict the proposed role of changes in arousal states as processes of sensitization, habituation and extinction have all been shown to contribute to the modulation of arousal (Lebestky et al., 2009). In terms of physiological mechanisms mediating site-specific sensitization, one possibility is wind-up sensitization, which is elicited by repeated application of noxious stimuli (Sandkühler, 2009). NMDA receptors are known to contribute to wind-up sensitization in mammals and repeated high-frequency stimulation of *Hirudo* nociceptors is known to produce NMDA receptor-dependent synaptic potentiation in nociceptive and non-nociceptive synapses (Franzen et al., 2023; Yuan and Burrell, 2019). Other physiological mechanisms potentially contributing to sensitization in *Hirudo* include decreases in GABAergic tone (disinhibition) (Baca et al., 2008; Paulsen and Burrell, 2022), endocannabinoid-based modulation (Jorgensen and Burrell, 2022; Wang and Burrell, 2018) and serotonergic modulation (Burrell and Sahley, 2005; Burrell et al., 2001; Ehrlich et al., 1992; Lockery and Kristan, 1991; Zaccardi et al., 2004).

It is also possible that habituation of the whole-body and local shortening reflexes contributes to this modulation of arousal. There was a decrease in shortening scores with repetitive stimulation, but whether this definitively represents habituation is speculative because the increasing level of evasion behaviors precludes accurate assessment of decreases in reflexive shortening. However, the role of habituation of the shortening reflexes makes sense from a functional perspective. There appears to be a hierarchy in behavioral responses to the needle pokes, with shortening reflexes predominantly observed in early trials, followed by evasion behaviors in later trials, regardless of whether the animals were ultimately in the early, intermediate or late evader category. The observed behavioral hierarchy is different from previous reports in *Hirudo* (Gaudry and Kristan, 2009; Misell et al., 1998), possibly due to differences in the type and pattern of stimulation in the current study. Nevertheless, reflexive shortening and evasion behaviors are mutually exclusive and distinct neural circuits mediate crawling versus withdrawal of the posterior sucker. It is possible that habituation of the shortening reflexes must occur first in order to ‘get out of the way’ of the evasion behaviors. For example, higher hierarchal behaviors may inhibit lower hierarchal behaviors (Kovac and Davis, 1977), but as the former habituate, their inhibitory influence on the latter may decrease. If simultaneous habituation of one behavior and sensitization of a second behavior is required for the observed increase in arousal, this would be an interesting application of the classic dual-process theory of habituation and sensitization (Groves and Thompson, 1970; Rankin et al., 2009).

An alternative interpretation is that the animals are learning to avoid nociceptive stimuli as a form of operant conditioning, similar to what has been reported in other invertebrates, e.g. mollusks, crustaceans and insects (Brembs, 2003; Elwood, 2019; Hawkins et al., 2006; Lovell and Eisenstein, 1982; Lukowiak et al., 1996; Magee and Elwood, 2013). Prior injury may enhance such learning as a result of increased arousal and/or sensitization. However, additional experiments are needed to support the idea that the increased evasions in *H. verbana* are due to operant learning.

In conclusion, repeated noxious mechanostimuli were shown to elicit a complex set of behavioral responses in *H. verbana*. Evidence suggesting that this represents the influence of arousal includes persistence of the behavior even when stimuli had not been immediately/directly applied, a multi-modal distribution of evasion strategies that potentially represents distinct internal motivational states, and the capacity to modulate the evasion strategy as a result of prior experience, i.e. injury. Increases in arousal in the context of nociception are often thought to reflect changes in the affective/emotional component of pain (Auvray et al., 2010), a concept that is usually not applied to invertebrates and is controversial. While we are not advocating that *H. verbana* has an emotional capacity for pain equivalent to that of mammals, it is possible that these arousal processes in the context of nociception represent an example of the ‘emotion primitives’ concept proposed by Anderson and Adolphs (2014). This idea states that there are fundamental behavioral elements that can be found across the animal kingdom that are not equivalent to human emotions but are constituents of those complex affective behaviors that likely have adaptive value. This is a concept that could be usefully applied to the study of pain-like behaviors or behavioral states in invertebrates and non-mammalian vertebrates (Crump et al., 2022; Sneddon, 2009). Looking forward, there are opportunities to address the neurophysiological mechanisms mediating arousal-based modulation in the *Hirudo* CNS. Command-like neurons that can elicit swimming or crawling have been identified in the leech head brain and in the penultimate posterior ganglion, the latter of which is in close proximity to where stimuli were applied in the present study (Esch et al., 2002; Mesce et al., 2008; Mullins and Friesen, 2012; Mullins et al., 2011). In addition, Kristan and colleagues have identified a ‘preparatory network’, a population of neurons in *Hirudo* that receive sensory input and are active prior to the neurons that mediate specific motor behaviors, e.g. for shortening, swimming and crawling (Frady et al., 2016). Perhaps this preparatory circuit contributes to arousal states, modulating behavioral choice and/or contributing to the scale or magnitude of the eventual behavioral response. Overall, there are clearly benefits in comparative studies of the biology and evolution of nociception and pain that can contribute to discovering and understanding conserved mechanisms for acute and chronic pain, which may lead to improved human and animal welfare.

## Supplementary Material

10.1242/jexbio.245895_sup1 Supplementary information
