# Supplementary material for: Repetitive nociceptive stimulation elicits complex behavioral changes in Hirudo: evidence of arousal and motivational adaptations
Source: J Exp Biol. 2023 Aug 15;226(16):jeb245895. doi: 10.1242/jeb.245895 (PMC10445732; doi:10.1242/jeb.245895)
Supplement: Supplementary information [file jexbio-226-245895-s1.pdf]

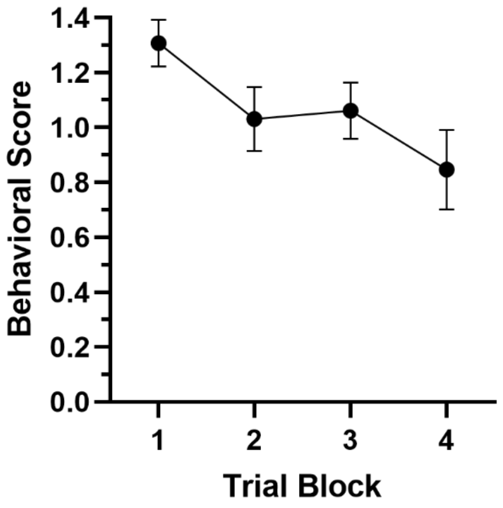

Fig. S1. Decreases in the shortening response in late evaders.

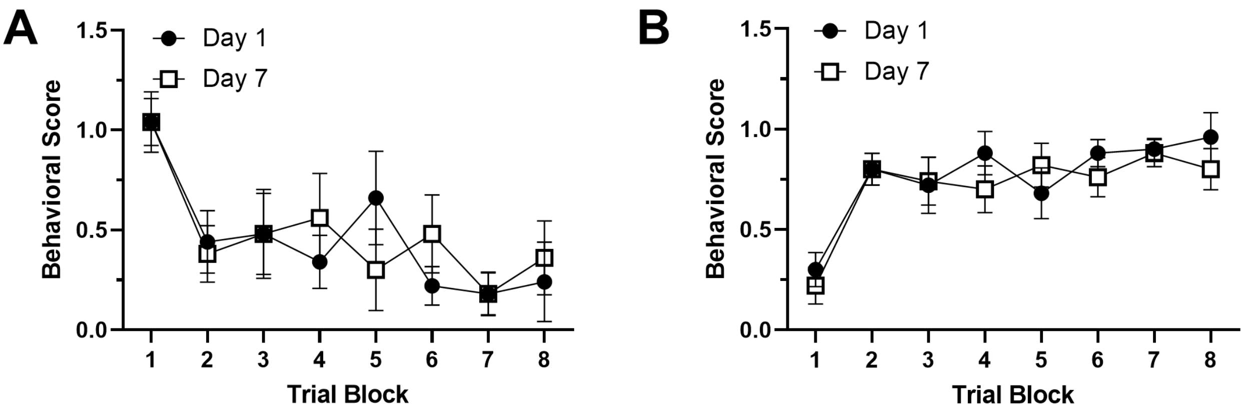

Fig. S2. Effect of retesting on shortening and evasion behaviors. *Hirudo* tested on day 1 and then re-tested on day 7 exhibited no changes in either habituation of withdrawal reflexes (A) or increases in evasion behaviors (B).

Journal of Experimental Biology • Supplementary information

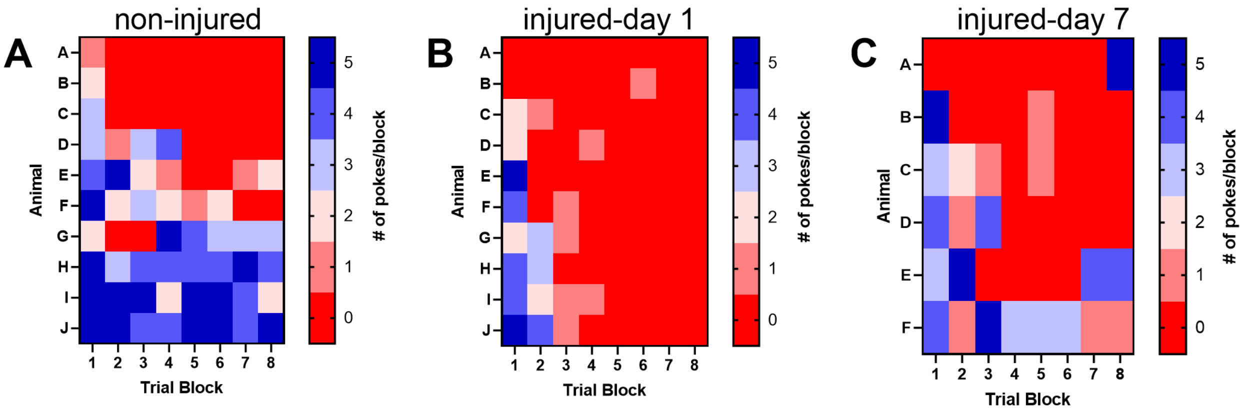

Fig. S3. Raster plot showing distribution of animal responses to needle pokes. Colors indicate the number of pokes/trial block, and fewer pokes indicate that the animal was engaged in evasion behaviors. (A) The subset of non-injured animals included in this analysis exhibited a full range of behavioral strategies similar to their parent group show in Fig. 2D; animals that received relatively few pokes and only early in trial blocks (early evaders, blue), those that received many pokes through all 8 trial blocks (later evaders, redd) and those that fell into the intermediate category. (B) Injured-day 1 animals as a whole received relatively few pokes in later trial blocks. (C) Most injured-day 7 animals received relatively few pokes late in testing, but there were some animals that received pokes throughout testing in this group.

Journal of Experimental Biology • Supplementary information
